# Supplementary material for: Saccharomyces boulardii (CNCM I-745) improves intestinal damage in sepsis by remodeling intestinal flora structure
Source: Front Cell Infect Microbiol. 2025 Jul 31;15:1602792. doi: 10.3389/fcimb.2025.1602792 (PMC12350329; doi:10.3389/fcimb.2025.1602792)
Supplement: Supplementary file 1 [file DataSheet1.zip › summary/summary/7_Differential_analysis/2_LEfSe_result/Group_DW0_vs_BW0_0.05_3.0/LEfSe_Group_DW0_vs_BW0.abund.pdf]

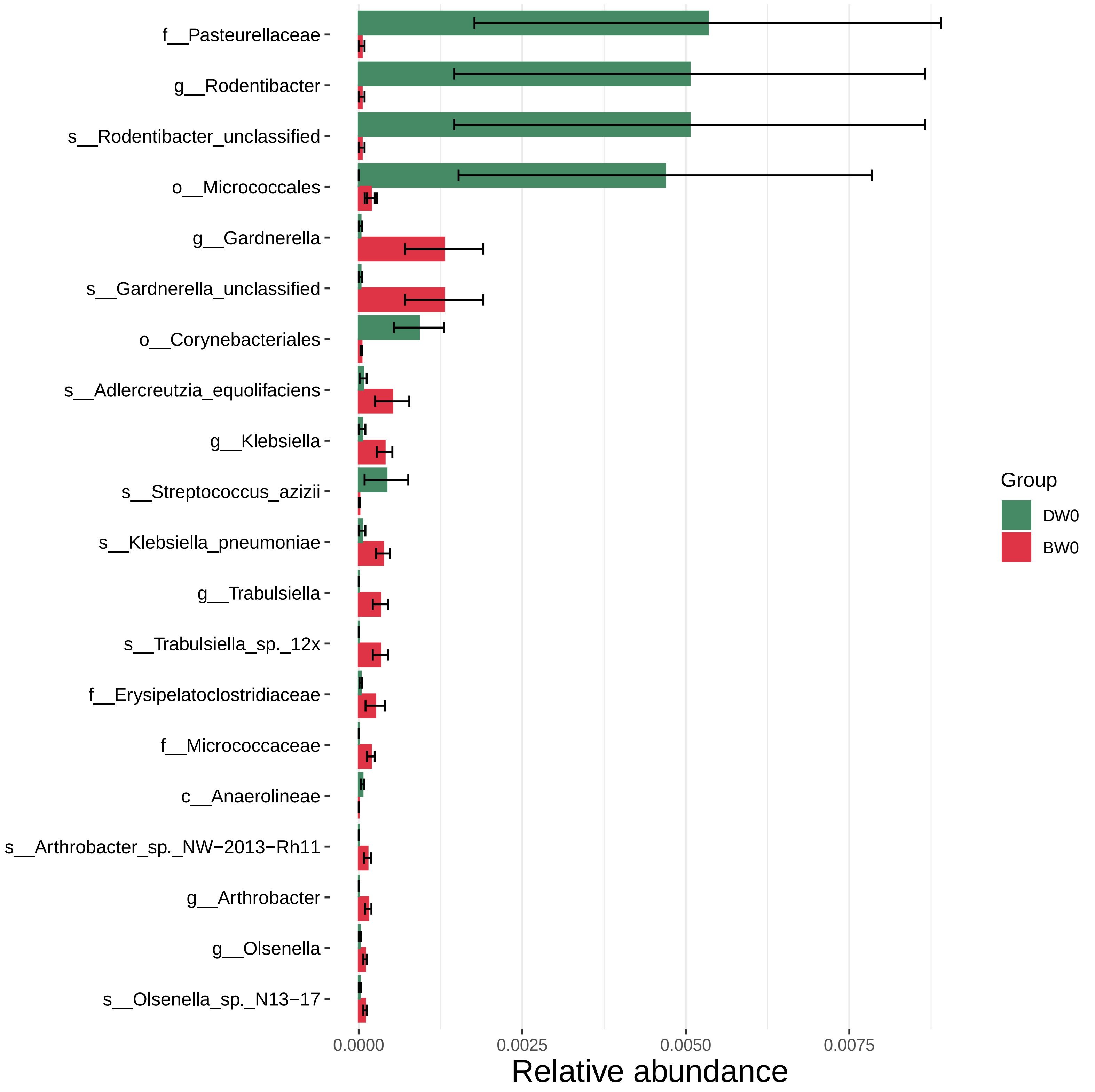
f__Pasteurellaceae
g__Rodentibacter
s__Rodentibacter_unclassified
o__Micrococcales
g__Gardnerella
s__Gardnerella_unclassified
o__Corynebacteriales
s__Adlercreutzia_equolifaciens
g__Klebsiella
s__Streptococcus_azizii
s__Klebsiella_pneumoniae
g__Trabulsiella
s__Trabulsiella_sp._12x
f__Erysipelatoclostridiaceae
f__Micrococcaceae
c__Anaerolineae
s__Arthrobacter_sp._NW−2013−Rh11
g__Arthrobacter
g__Olsenella
s__Olsenella_sp._N13−17
0.0000
0.0025
0.0050
0.0075
Relative abundance
Group
DW0
BW0
